# Effective connectivity between superior temporal gyrus and Heschl’s gyrus during white noise listening: linear versus non-linear models

**DOI:** 10.2349/biij.8.2.e13

**Published:** 2012-04-01

**Authors:** KA Hamid, AN Yusoff, MZA Rahman, M Mohamad, AIA Hamid

**Affiliations:** 1 School of Diagnostic Sciences and Applied Health, Faculty of Health Sciences, Universiti Kebangsaan Malaysia, Kuala Lumpur, Malaysia; 2 Radiology Services, National Heart Institute, Kuala Lumpur, Malaysia; 3 Department of Neurosciences, School of Medical Sciences, Universiti Sains Malaysia, Kubang Kerian, Malaysia

**Keywords:** fMRI, dynamic causal modelling (DCM), Bayesian Model Selection (BMS), non-linear DCM

## Abstract

**Purpose:**

This fMRI study is about modelling the effective connectivity between Heschl’s gyrus (HG) and the superior temporal gyrus (STG) in human primary auditory cortices.

**Materials & methods:**

Ten healthy male participants were required to listen to white noise stimuli during functional magnetic resonance imaging (fMRI) scans. Statistical parametric mapping (SPM) was used to generate individual and group brain activation maps. For input region determination, two intrinsic connectivity models comprising bilateral HG and STG were constructed using dynamic causal modelling (DCM). The models were estimated and inferred using DCM while Bayesian Model Selection (BMS) for group studies was used for model comparison and selection. Based on the winning model, six linear and six non-linear causal models were derived and were again estimated, inferred, and compared to obtain a model that best represents the effective connectivity between HG and the STG, balancing accuracy and complexity.

**Results:**

Group results indicated significant asymmetrical activation (*p*_uncorr_ < 0.001) in bilateral HG and STG. Model comparison results showed strong evidence of STG as the input centre. The winning model is preferred by 6 out of 10 participants. The results were supported by BMS results for group studies with the expected posterior probability, *r* = 0.7830 and exceedance probability, *ϕ *= 0.9823. One-sample *t*-tests performed on connection values obtained from the winning model indicated that the valid connections for the winning model are the unidirectional parallel connections from STG to bilateral HG (*p* < 0.05). Subsequent model comparison between linear and non-linear models using BMS prefers non-linear connection (*r* = 0.9160, *ϕ *= 1.000) from which the connectivity between STG and the ipsi- and contralateral HG is gated by the activity in STG itself.

**Conclusion:**

We are able to demonstrate that the effective connectivity between HG and STG while listening to white noise for the respective participants can be explained by a non-linear dynamic causal model with the activity in STG influencing the STG-HG connectivity non-linearly.

## Introduction

Functional magnetic resonance imaging (fMRI) is one of the many modalities used in neuroscience studies to detect brain activation resulting from a performed task [1]. It basically measures the haemodynamic responses related to neuronal activity in human as well as animal brains. These responses are based on changes in blood flow, volume, and oxygenation and are commonly known as blood-oxygenation level dependent (BOLD) [1].

Compared to fMRI studies done on other cortical areas, the study on human auditory cortices has lagged behind. This is probably due to the problem of background noise generated by the magnetic resonance scanner during the scan, which can cause unnecessary additional activation in the auditory areas, contributing to the existence of artifacts on the functional images. Therefore, sparse or silent fMRI techniques have been developed to overcome these artifacts [2].

Nevertheless, the functional specialisation of human auditory cortices has been established and reported in many studies [3–5]. Heschl’s gyrus (HG), for example, has been known to be the primary processing centre for auditory stimuli. Previous studies reported bilateral activation of HG in normal hearing participants as a result of listening to simple tones [6, 7]. On the other hand, a study using EEG reported that white noise evoked responses in the primary auditory cortices (PAC), secondary auditory cortices (SAC), plenum temporal (PT), insula, and sulcus between the PAC and PT [8].

Previous studies of the human auditory system have mostly focused on the functional specialisation of the brain in response to auditory tasks only, rather than the dynamic interaction or connectivity between the activated brain regions [9]. This is termed “effective connectivity” and can be understood as the influence that a neuronal system exerts over another at the synaptic or cortical level [10].

The effective connectivity between activated areas in the brain can be determined using dynamic causal modelling (DCM) by taking into account the posterior probability of the occurrence of the interaction between any two activated areas. DCM is a standard non-linear system identification approach and treats the human brain as an input-state-output system [10]. The parameters involved are estimated by measuring the response between the activated brain areas [10]. Three types of variables in DCM are the input variables that encode experimental manipulation; the output variables characterising the regional haemodynamic responses from each region; and the state variables that represent the neuronal activity and biophysical variables that transform neuronal activity into a haemodynamic response [11].

In DCM, the dynamics between interacting neuronal populations are modelled using a bilinear state equation [10],

(1) $$\frac{dx}{dt}=\left(A+\sum_{i=1}^{m}u_{i}B^{\left(i\right)}\right)x+Cu$$

Three sets of parameters are estimated in Equation (1) for bilinear causal models, which are (i) the intrinsic connection strength between regions in the absence of any external experimental input matrix ; (ii) the modulatory input that changes the intrinsic connection strength induced by experimental input ***u_i_***, matrix ***B***, and (iii) the direct influence of a stimulus on a given region, matrix ***C*** [10]. Integrating Equation (1) over time (*t*) gives predicted neuronal dynamics that enter a model of haemodynamic response to give predicted BOLD response parameters. These neuronal dynamics are determined by experimental manipulations and enter the model in the form of external inputs, *u.*

Bilinear models of effective connectivity have limitations [12, 13]. Firstly, the neuronal origin of the modulatory influence is not specified, unless the origin of the neuronal population that modulates a connection in a cortical network is the question of interest. Secondly, since they are mediated by non-linear effects at the level of single neuron, the fast changes in effective connectivity are not appropriately presented by a bilinear framework. Thirdly, in a bilinear model, the strength of any given connection is independent of the activity in remote neuronal populations. In other words, a bilinear model is unable to identify whether the connectivity modulation between two brain areas is caused by any other neuronal population or not [12, 13].

In the present study, the connectivity between the activated brain regions during a white noise listening task was investigated. The HG and STG of the right and left hemispheres were selected as the regions of interest and the intrinsic couplings connecting these four regions were then assessed using DCM. The flexibility of the bilinear and non-linear models was then tested to best describe the effective connectivity between those areas.

## Methods

### Participants

Ten healthy male participants were recruited for this study. All participants were interviewed on their state of health and were screened for middle ear conditions and hearing levels in the frequency range of 800Hz to 2500 Hz. The participants were given informed consent and screening forms as required by the institutional ethics committee (IEC).

### fMRI scan, stimulus, and paradigm

**Figure 1:**
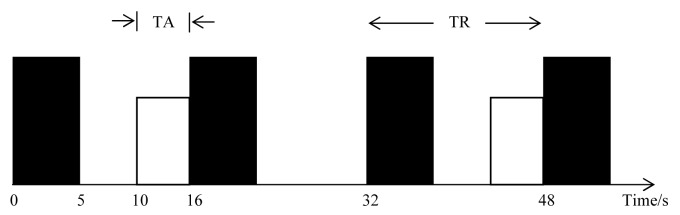
Timing diagram of the silent fMRI paradigm used in this study. Dark boxes represent the 5-s EPI measurements while white boxes represent the 6-s stimulus (white noise) delivery.

The fMRI scans were carried out using a 1.5-tesla MRI system (Siemens Magnetom Avanto). The scan covers the bilateral auditory cortex of the brain. Participants were asked to lie supine on the MRI couch. The head coil was used for the delivery of the radiofrequency (RF) pulses. All participants were asked to pay attention to the given stimulus and to respond by pressing the squeeze bulb immediately after hearing the stimulus. White noise was used as the auditory stimulus and was set at an intensity of 70 dB higher than the hearing level of a normal individual. The stimulus was presented binaurally to the participants via headphones. A silent imaging paradigm (Figure 1) was used in this study to minimise the artifacts on the images caused by the sound of the fMRI scanner [2]. This is so the resulting haemodynamic response could be measured accurately without interference from the haemodynamic response from the echo planar imaging (EPI) scan. White noise was alternately delivered in durations of 6s prior to EPI scanning (dark box). In Figure 1, only four EPI measurements and two stimulus deliveries are shown. The repetition time (TR), which is the time interval between one particular slice of a measurement and the same slice of the following measurement, was 16 s, while the acquisition time (TA), which is the time taken for a complete scan of the whole brain volume of interest, was 5 s. A long TA was used in this silent imaging paradigm due to the fact that the haemodynamic response in the auditory cortex will peak in 10 s from the time of stimulus delivery, as reported in a previous study [14]. The time interval from each stimulus to the next was 32 s.

### Post-processing of data

The fMRI data were analysed using MATLAB 7.4 – R2008a (Mathworks Inc. MA, USA) and Statistical Parametric Mapping (SPM8) (Functional Imaging Laboratory, Wellcome Department of Imaging Neuroscience, Institute of Neurology, University College London) packages. A conventional analysis based on the general linear model was used to generate brain activation in the regions of interest using the statistical T-test for each voxel. Individual participant analysis was performed at the corrected significance level (*α*_corrected_) of 0.05. For the group analysis, the random effect analysis (RFX) was used and statistical inferences were made at the significance level, *α *= 0.001, uncorrected for multiple comparisons between participants. Conjunction analysis was also done to assess the common activated areas in all participants by specifying contrasts separately for each participant under study. Statistical inference for conjunction analysis was made at *α *= 0.1, uncorrected.

### Dynamic causal modelling (DCM)

The effective connectivity among the regions of interest (ROIs) was studied using dynamic causal modelling (DCM). The four brain regions that play a central role in auditory stimulus processing were considered, i.e. the right and left HG, and right and left STG. These two areas comprise the PAC. The anatomical location of the activation peak for these regions having significant voxels at uncorrected *α* < 0.001 was confirmed using the ROI masks based on the Talairach Daemon together with the WFU PickAtlas software [15]. These four ROIs were co-registered onto each individual based on the coordinates of the activation peak obtained in the group random effects analysis (RFX). The centre of each ROI (defined as a sphere of 4 mm radius) was constrained so that its displacement from the peak activation in RFX lies within 16-mm distance, which is twice the full-width-at-half-maximum (FWHM) of the smoothing kernel. This approach is similar to that used in a previous study [9].

**Figure 2:**
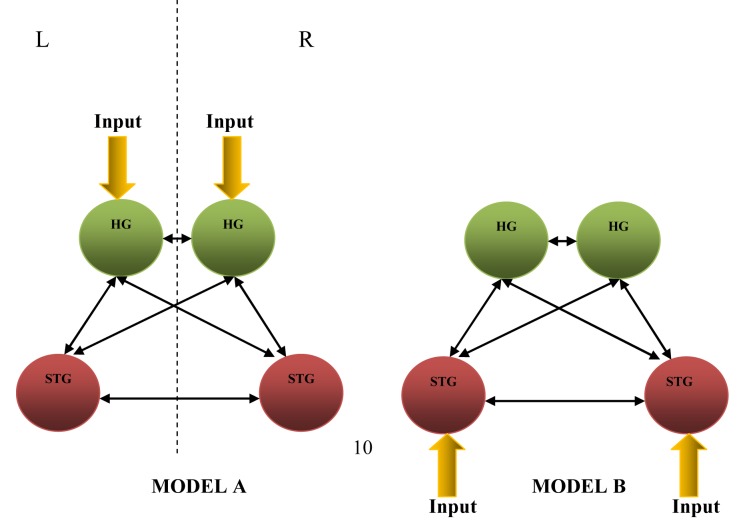
Fully-connected non-modulated models for input region determination for the first stage of DCM analysis (L – left hemisphere, R – right hemisphere).

The coordinates extracted earlier from RFX statistical parametric maps were used in the first stage of DCM analysis, i.e. the determination of the input region(s). Two fully-connected non-modulated models (Figure 2), constructed based on the four extracted coordinates, were tested on each participant. The first model (Model A) receives inputs from bilateral HG, while the second model (Model B) receives inputs from bilateral STG. The two models were entered into DCM and estimated for each participant to obtain the influence of the direct inputs to the system, which are the values of matrix C, and strength of intrinsic connections or couplings between the modelled regions, which are the values of matrix A [10], see also Equation (1). These two models were further compared using Bayesian Model Selection (BMS). To test for the most probable existence of connections between these four regions, one sample *t*-tests were done against zero on each of the 12 connections on all the participants.

The second stage of model construction and comparison was to determine whether the preference for the optimal connectivity model is linear or non-linear. For each participant, having the input centre determined earlier (which will be shown in the following section as propagating through bilateral STG), six bilinear and six non-linear causal models were constructed, consisting of similar patterns of intrinsic connections between those same four coordinates. What distinguishes these two groups of models is the state of input that alters or modifies the strength of connections between the neuronal populations involved, whether by external modulatory input (bilinear causal model) or by region (non-linear causal model) itself. These models were then estimated using DCM and compared using BMS to determine the most probable model among each of bilinear and non-linear model groups. The winning models from each group were then compared using BMS to determine the optimum model that best describes the state of connectivity between the activated neuronal populations as a bilinear or non-linear interacting network. The approach used in the second stage of model construction and comparison is in accordance with the approach used in a previous study [12].

## Results

### Participants

The participants were confirmed to be healthy. Their average age 25.5 ± 0.3 years. Pure-tone audiometry (PTA) testing performed on participants confirmed that all participants had normal hearing with no neurological disorders.

### Functional specialisation

At *α* = 0.05 (corrected for multiple comparisons), a dominant right-sided activation was consistently observed in all participants. All participants showed significant activation in bilateral STG (*p* < 0.05), particularly in the PAC. At least five participants showed significant common activation (*p* < 0.05) in HG, inferior frontal gyrus (IFG), supramarginal gyrus, and left rolandic operculum.

**Figure 3:**
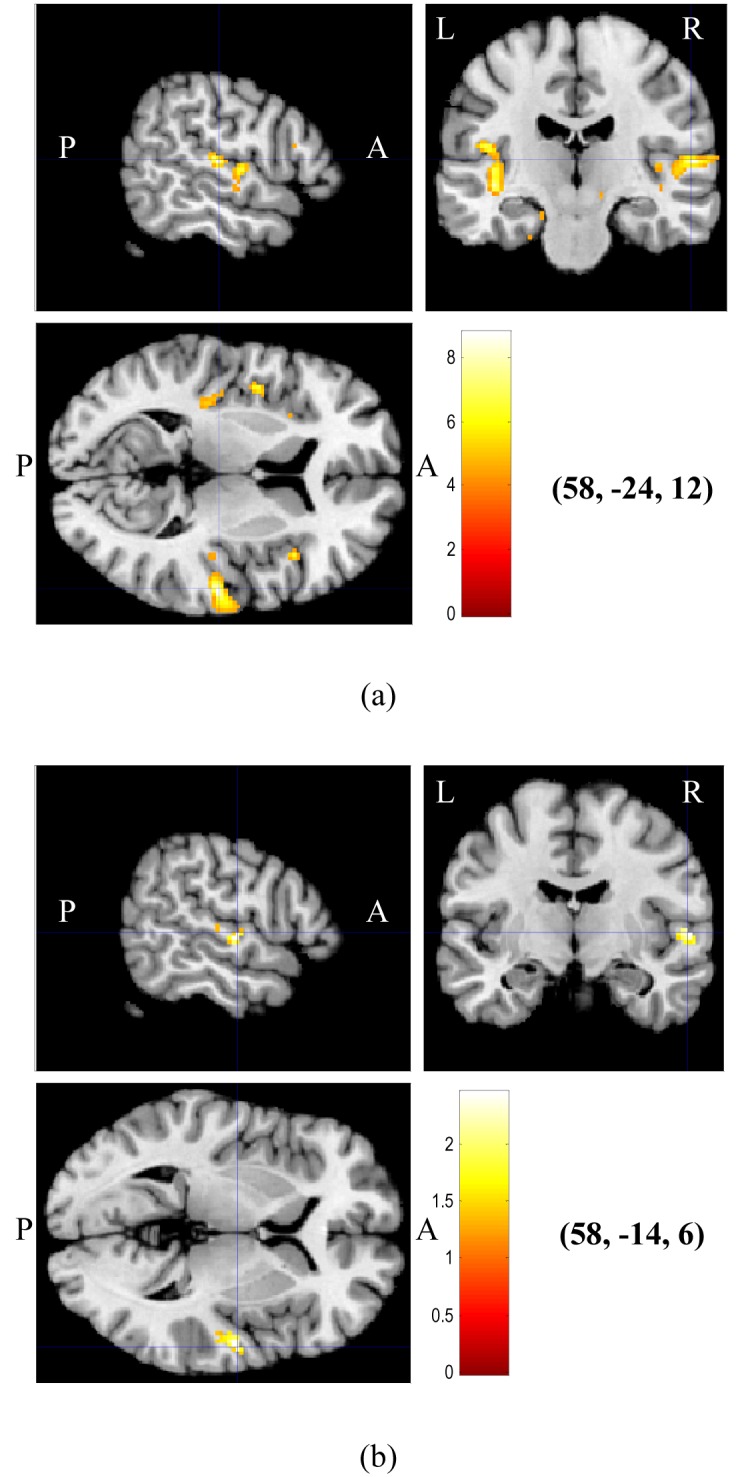
Statistical parametric maps (SPMs) showing brain activation overlaid onto structural brain images, obtained from (a) RFX on all participants at uncorrected *α* = 0.001, and (b) conjunction analysis on all participants at uncorrected *α* = 0.1. The coordinates of maximum intensity indicated by the crossing hairlines are shown in brackets. Colour codes represent increasing *t* values from red to white (A – anterior, P – posterior, L – left hemisphere, R – right hemisphere).

Table 1 shows the MNI coordinates at the point of maximum intensity in each respective cluster and the anatomical area in which the point of maximum intensity in brain activation due to white noise listening occurs. The data were obtained from the RFX [16] statistical parametric maps (SPMs). The uncorrected *p*-values shown in the table are derived based on the set-level (number of activated regions), cluster-level (number of activated voxels), and voxel-level inferences (the *p* value for each voxel within the cluster) [16]. At uncorrected *α* = 0.001 and at a spatial threshold of 10 voxels, nine significant clusters survived the cut-off. There were a total of 1023 activated voxels (*t* > 4.30), with 674 voxels in the right hemisphere while the remaining 349 voxels were activated in the left. There were a total of 771 activated voxels activated in the primary auditory area, including STG and HG. Using a MATLAB-based PickAtlas toolbox (Wake Forest University, North Carolina, USA) [15] for anatomical interpretation, the right PAC consisted of 452 voxels (58.6%) while the left PAC contained a number of 319 voxels (41.4%). Unilateral activation occured in the left rolandic operculum, right cerebellum, right IFG (pars opercularis), right insula, right middle frontal gyrus (MFG), right vermis, and left parahippocampus. The results obtained from conjunction analysis indicate a total of 35 activated voxels in the right STG, two activated voxels in the left STG, and two activated voxels in the left insula. The main cluster has 12 activated voxels (*t* > 1.28) with the point of maximum intensity at (58, −16, 0) representing the right STG. The SPM results were generated at *α* = 0.1 (uncorrected for multiple comparisons). Figure 3 shows the SPMs obtained from RFX and conjunction analyses for comparison. For both results, the coordinates with the highest *t*-value were found to represent the right STG.

**Table 1:** Statistical data, Tailarach-MNI coordinates (*x*, *y*, *z*), and the respective anatomical areas obtained from RFX on 10 participants at *α*_uncorrected_ = 0.001 thresholded at 10 voxels.

**Cluster-level**			**Voxel-level**		***x*, *y*, *z* (mm)**			**Anatomical area**
**Cluster**	***p*_uncorrected_**	**No. of activated voxels**	***p*_uncorrected_**	***t*-value**				
1	< 0.001	452	< 0.001	8.78	58	−24	12	Right superior temporal gyrus
			< 0.001	7.41	56	−14	8	Right superior temporal gyrus
			< 0.001	7.25	44	−20	−2	Right superior temporal gyrus
2	< 0.001	319	< 0.001	8.18	−44	−20	−2	Left superior temporal gyrus
			< 0.001	6.91	−42	−22	8	Left superior temporal gyrus
			< 0.001	6.78	−46	−24	20	Left rolandic operculum
3	0.001	110	< 0.001	7.74	28	−52	−32	Right cerebellum
			< 0.001	6.14	26	−58	−26	Right cerebellum
4	0.005	75	< 0.001	6.69	40	14	10	Right opercular inferior frontal gyrus
			< 0.001	5.72	42	6	6	Right insula lobe
5	0.249	10	< 0.001	6.30	32	48	30	Right middle frontal gyrus
6	0.208	12	< 0.001	5.16	−20	4	−16	Left olfactory lobe
7	0.138	17	< 0.001	5.04	6	−58	−14	Vermis
			< 0.001	4.68	−2	−64	−16	Vermis
8	0.128	18	< 0.001	4.84	−16	−28	−14	Left parahippocampus
			< 0.001	4.47	−20	−18	−20	Left parahippocampus
9	0.249	10	< 0.001	4.73	42	14	−12	Right IFG operculum
			< 0.001	4.53	50	14	−10	Right IFG operculum

### Connectivity results

In this study, all the ten participants showed significant (*p* < 0.001) activation in bilateral HG and STG. As the respective individual ROI coordinates did not violate DCM requirements mentioned in the methods section, all the participants were included in the analysis of connectivity.

Group BMS results for input determination evoked by white noise for the ten participants were obtained by RFX for BMS [17]. Table 2 shows the Dirichlet parameter estimate (*α*_d_), expected posterior probability (<*r*>) and exceedance probability (*ϕ*) for Models A and B obtained from BMS. The results showed evidence of Model B’s superiority as compared to Model A (See Table 2 and Figure 2), indicating the right and left STG as the most probable input centre during white noise listening. The parameters *α*_d,_ <*r*> and *ϕ* will be defined in the discussion section.

**Table 2:** The Dirichlet parameter estimates (*α*_d_), expected posterior probability (<*r*>) and exceedance probability (*ϕ*) for both Models A and B obtained from BMS.

	**Model A** **(Input at HG)**	**Model B** **(Input at STG)**
*α_d_*	2.6036	9.3964
<*r*>	0.2170	0.7830
*ϕ*	0.0177	0.9823

The results obtained from one-sample *t*-testing of the intrinsic connection values from the preferred fully-connected Model B (Figure 2) for all the participants are tabulated in Table 3 with the respective *t*- and *p*-values. The *p*-value mentioned here is the probability of the connections to be zero. As can be seen, only some of the connections were found to be significant (*p* < 4.167 × 10^−3^ = *α *= 0.05/12 connections = with 95% CI). However, a statistically significant connection can only be accepted if its posterior probability (*P*) is relatively high with a value equal or greater than 0.9 [10].

**Table 3:** The input at bilateral STG, intrinsic connectivity between bilateral HG and STG, and their statistics based on Model B averaged over 10 participants (*P* – posterior probability, SD – standard deviation, R(STG) – right superior temporal gyrus, L(STG) – left superior temporal gyrus, R(HG) – right Heschl’s gyrus, L(HG) – left Heschl’s gyrus).

**Input/Hz**		**Connectivity/Hz**	***P***	**SD**	***p***	***t***
Input at (L)STG	0.1356	-	1.0000	0.1131	3.333 × 10^−4^	3.791
Input at (R)STG	0.2551	-	1.0000	0.0950	0.000	8.495
(L)HG to (L)STG	-	0.0018	0.8138	0.0129	5.617 × 10^−2^	0.435
(L)HG to (R)HG	-	0.0145	0.8492	0.0250	8.250 × 10^−3^	1.838
(L)HG to (R)STG	-	0.0101	0.5235	0.0204	1.275 × 10^−2^	1.562
**(L)STG to (L)HG**	**-**	**0.0973**	**1.0000**	**0.0974**	**1.000 × 10^−3^**	**3.158**
**(L)STG to (R)HG**	**-**	**0.0801**	**1.0000**	**0.0856**	**1.333 × 10^−3^**	**2.959**
(L)STG to (R)STG	-	0.0238	0.5243	0.0321	3.667 × 10^−3^	2.346
(R)HG to (L)HG	-	0.0199	0.9322	0.0283	4.417 × 10^−3^	2.231
(R)HG to (L)STG	-	0.0011	0.8553	0.0119	6.500 × 10^−2^	−0.288
(R)HG to (R)STG	-	0.0042	0.7825	0.0136	2.908 × 10^−2^	0.988
**(R)STG to (L)HG**	**-**	**0.1716**	**1.0000**	**0.1432**	**3.333 × 10^−4^**	**3.790**
(R)STG to (L)STG	-	0.0233	0.9134	0.0395	8.000 × 10^−3^	1.860
**(R)STG to (R)HG**	**-**	**0.1339**	**1.0000**	**0.1032**	**2.500 × 10^−4^**	**4.101**

**Figure 4:**
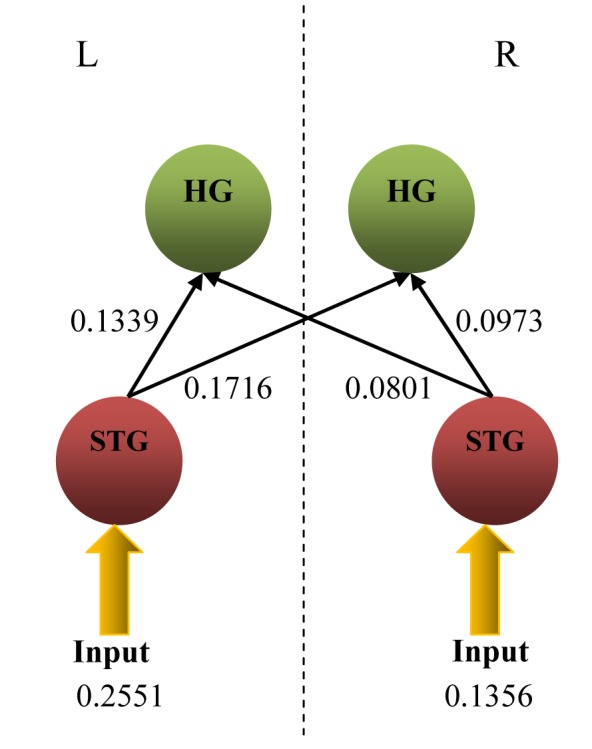
The most probable linear dynamic causal model (DCM) for listening to white noise with input and connection values obtained from DCM and statistical analyses (L –left hemisphere, R – right hemisphere).

From Table 3, the significant connections from DCM analysis and the *t*-test that are considered for the construction of the most probable model are (L)STG → (L)HG, (L)STG → (R)HG, (R)STG → (L)HG and (R)STG → (R)HG (in bold). The model is shown in Figure 4. The average values (in Hz) for the accepted input and connections are also given in Figure 4.

### Bilinear dynamic causal models

**Figure 5:**
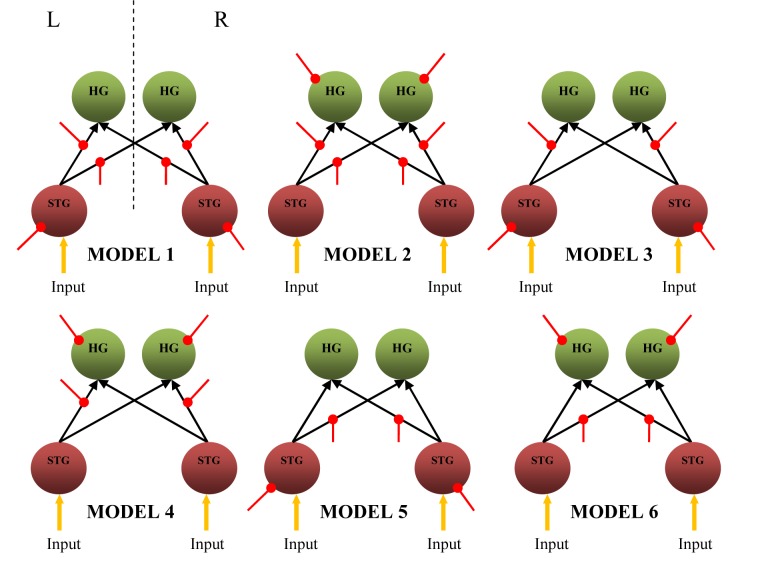
Six bilinear causal model having connections and regions influenced by external modulation (red line) (L – left hemisphere, R – right hemisphere, external modulation).

BMS for group studies on the six bilinear causal models (Figure 5) constructed based on the winning model (Figure 4) has indicated Model 1 as the preferred bilinear model, with Dirichlet parameter estimates, α*_d_* = 7.8684, expected posterior probability, <*r*> = 0.4918 and exceedance probability, *ϕ* = 0.9068). Model 1 assumes that noise has a direct influence on bilateral STG and modulates the strength of all the four intrinsic connections.

### Non-linear dynamic causal models

**Figure 6:**
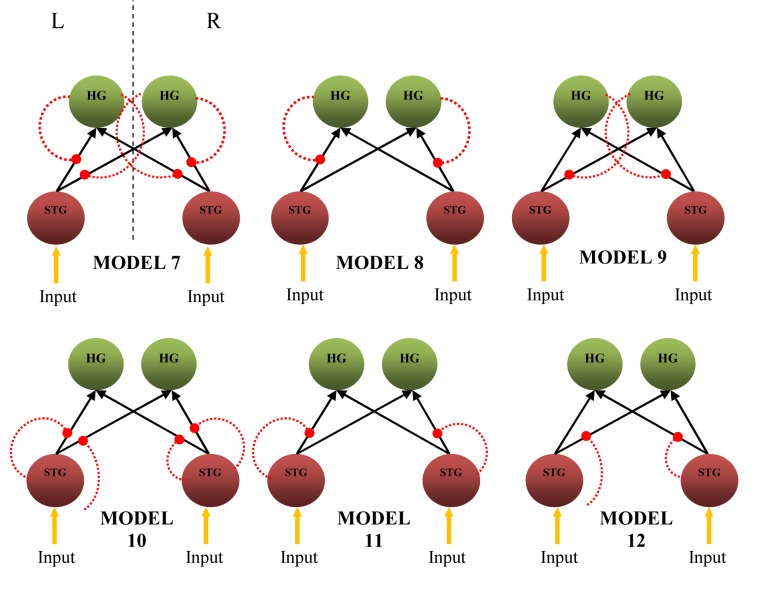
Six nonlinear causal models having connections gated by the activity in the HG and STG (red dotted line) (L – left hemisphere, R – right hemisphere, region-gated modulation, HG – Heschl’s gyrus, STG – superior temporal gyrus).

Figure 6 shows the non-linear extension of dynamic causal models. The models assume that the effective connectivity in a neuronal network can be explained by non-linear mechanism types. Non-linear DCM was suggested based on the idea that the intrinsic connection between several brain regions could be gated by the activity in another activated brain region, resulting in non-linear type connectivity [12]. A comparison of these models by BMS using RFX analysis indicates Model 10 as the most probable model, with a non-linear modulation by each STG on intrinsic connections from ipsilateral STG to both ipsilateral and contralateral HG in both hemispheres. The results obtained from the comparison are *α_d_*= 8.0627, <*r*> = 0.5039, and *ϕ* = 0.9494.

**Figure 7:**
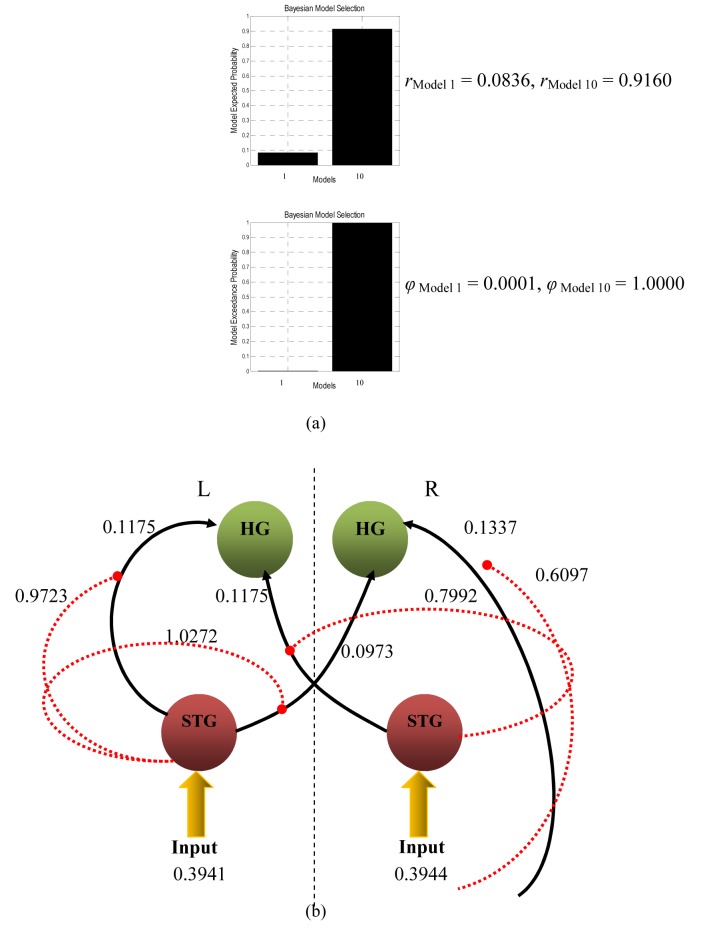
(a) Bayesian Model Selection comparison results between bilinear (Model 1) and non-linear (Model 10) models, and (b) the most probable effective connectivity model for listening to white noise, balancing accuracy and complexity (all values are in Hz).

Taking the most probable bilinear and non-linear causal models for further analysis, Model 1 of Figure 5 and Model 10 of Figure 6 were compared using BMS. Model 10 was finally found to be superior to Model 1 and resulted in *α*_d_, <*r*>, and *ϕ* of 10.9978, 0.9165, and 0.9995, respectively. The histogram obtained from BMS comparison results are depicted in Figure 7(a). It can be said that the connections from STG to bilateral HG are non-linear and most unlikely to depend on external modulation but on the activity in STG itself. The schematic representation of the model is illustrated in Figure 7(b).

## Discussion

### Silent fMRI paradigm (sparse temporal sampling)

A silent fMRI paradigm was used in this study due to its relatively high sensitivity in detecting brain activation and to avoid interference from the scanner sound with the auditory paradigm [21, 22]. According to [22], a silent fMRI paradigm is the method of choice if the experimental hypothesis concerns the functional network containing PAC. They also recommend a silent fMRI paradigm if the interpretation of brain activation is within the auditory cortex and is based on auditory selective attention. In a silent imaging paradigm, there is a finite time interval between the stimulus delivery and the peak of haemodynamic response in the brain depending on the task being given. For example, in visual and motor task responses, the brain haemodynamic responses normally peak at the 5^th^ to 8^th^ second from the time of stimulus delivery. In the case of auditory tasks, a longer time is needed for the haemodynamic response to peak, i.e. around 10.8 s [14, 20]. Thus, we believe the silent fMRI paradigm with a long TA (5 s) used in this study is capable of capturing the haemodynamic response generated in the auditory cortices after the stimulus (white noise) was given to the participant.

### Functional specialisation

The PAC lies in the posterior half of the STG, occupies most of the transverse temporal gyrus or HG [21], and is known to process auditory information [22]. The use of auditory stimuli in this study specifically activated parts of the temporal lobe in these two areas in at least five of the participants (*p*_corrected_ < 0.05). The activation in HG is also consistent with the established mechanism of HG involvement in the early processing of auditory information. White noise used in this study was also found to evoke activation in the posterior part of the STG as well. According to previous studies [20, 23], this region will be activated whenever sound of a broad frequency spectrum, such as white noise, is used as a stimulus. However, from analyses of individual participants, more than half of the participants evoked activation in non-primary auditory areas, such as IFG, supramarginal gyrus and left rolandic operculum. This is consistent with previous findings [20, 23] due to the fact that the frequency distribution of white noise is broad and white noise is known to be able to evoke activation in a large neuronal population of the human brain, even in these non-primary auditory areas.

The RFX done on this group of participants considers the randomness of different responses from participant to participant and takes within- and between-participant variability into account. Group RFX results show bilateral activation with a stronger activation in the right hemisphere, indicated by a higher number of activated voxels in the right STG than in the left. This is consistent with previous findings [24, 25], which show that the right temporal regions are dedicated to non-verbal auditory processing, such as pure tones and noise. The bilateral activation of the posterior STG is related to the broad frequency spectrum of the white noise used [16]. Furthermore, the STG is more responsive to complex auditory stimuli [7, 24] as compared to HG, which is relatively sensitive towards pure tone levels [6]

The common activated area due to listening to white noise in all participants as revealed by conjunction analysis lies in the right posterior STG at coordinates (58, −14, 6). These coordinates seem to shift by 11.6 mm from the coordinates of maximum intensity obtained from RFX, which are (58, −24, 12) but still in the same anatomical region. As mentioned earlier, the STG is a region in the PAC and is responsible for processing complex auditory stimuli [7, 24]. The occurrence of maximum intensity in the same anatomical region, as revealed by RFX and conjunction analysis, supports the fact that this region (the STG) is responsible for the reception of non-verbal auditory stimulus [6, 11].

### Dynamic causal modelling

The analysis of functional specialisation alone is insufficient to gain information about the dynamic of interaction between the activated brain regions. The dynamic of interaction includes connectivity between regions, such as in their direction and strength. The types of brain connectivity in a neuronal network – whether they are linear, bilinear [10] or non-linear [12] and whether they are single-state or two-state [10, 26] – are of considerable concern in any dynamic study of human brain function. Therefore, a detailed mechanistic approach developed to investigate the dynamic characteristics of the brain, dynamic causal modelling (DCM) [10] in particular, was used in this study to explore the connectivity among the activated brain regions and identify how they are influenced by external modulation.

DCM treats the brain as a dynamic input-state-output system. It is basically a non-linear system identification procedure and uses Bayesian parameter estimation to draw inferences about the effective connectivity between different regions in the brain [10]. In the present study, we investigated the effective connectivity between auditory areas of the right and left brain hemispheres. The left and right STG and HG are extracted as ROIs for this purpose. The justification is that these areas are responsible for sound processing, in which HG is known to be responsible for simple monotones, while the posterior STG is responsible for complex sounds. The first step in connectivity analysis involves determination of the input region, which is optimally selected between HG and the STG. DCM uses a fully Bayesian approach in estimating and selecting the optimal model among competing models, i.e. BMS [17]. Basically, BMS is fully statistic in approach and computes an approximation to the model evidence *p*(*y|m*), which is the probability of obtaining the data *y,* given the model *m* [27]. It quantifies the properties of a good model by explaining the data as accurately as possible and has minimal complexity [9, 28]. In other words, BMS determines the model that provides the best balance between the accuracy of fit and the complexity of the model [26]. The constructed models are usually compared by optimising the probability of conditional density for each model, given its respective log-evidences [17]. This Bayesian approach is robust since it accounts for presence of group heterogeneity or outliers [17].

Due to the binaural delivery of auditory stimuli, we assume that the external input enters the auditory cortex bilaterally. Two fully connected non-modulated models were constructed for comparison (Figure 2) to minimise any variability in either model, since the objective of this first stage of comparison is to determine the input region. Comparison using BMS shows a preference for the STG as the input region (Model B), with higher Dirichlet parameter estimates (*α*_d_), expected posterior probability (*<r>*) and exceedance probability (*ϕ*) (See Table 2). Dirichlet parameter estimates, *α*_d_, measure the effective number of participants for which a given model generates the observed data, while exceedance probability, *<r>*, is the probability that a model *k* generated the data for a randomly selected participant. The exceedance probability, *ϕ*, is the probability for a model *k* to give the observed experimental data compared to all the other models [17]. When *ϕ_k_* = 0.95, we can say that one is 95% sure that model *k* is favoured with a greater posterior probability (*<r>*) than any other competing model.

To determine the significant intrinsic connections from the winning full-connectivity model (Model B of Figure 2), the intrinsic coupling values (element of matrix **A**) and the input values (element of matrix **C**) in the bilinear neuronal state equation are considered. As can be seen from one-sample *t*-test results (Table 1), which show the average extrinsic input and intrinsic couplings, only some connections are found to be significant (*p *< 4.167 × 10^−3^ = *α* = 0.05/12 connections with a 95% CI), judging from the *p*-values and effect size (*t*-value). The average intrinsic connection value was tested against the condition of no connection, i.e. ‘0’.

One-sample *t*-test results obtained on the input and intrinsic connection values of each model through all the participants show four significant intrinsic connections with respective posterior probability of more than 0.9 [10], which are (L)STG→(L)HG; (L)STG→(R)HG; (R)STG→(L)HG; and (R)STG→(R)HG (Figure 4). These connections are written in bold-face in Table 3. It can be clearly seen that the four significant connections indicated the largest *t* values and have large effective connectivity values (in Hz) with the posterior probability of 1.000 as compared to other connections. The extrinsic inputs that were found to enter through the bilateral STG have also indicated high posterior probability (1.000) and *t* values. However, the fact that the input obtained from this study entered the auditory cortices through the bilateral STG opposes many previous reports using complex tones [11, 29, 30]. These studies reported HG as the input region. However, the posterior STG has also been reported to process white noise [31–33], in a similar fashion to HG for simple tones. Thus, it is possible to conclude that white noise, acting as the perturbation input, entered through the bilateral STG and induced driving intrinsic connections from these regions to the ipsilateral and contralateral HG bilaterally.

### Bilinear dynamic causal models

Model 1 in Figure 5 is the most probable bilinear model obtained from model comparison using BMS. It has external modulation on all the four intrinsic connections and also on bilateral STG. The modulation of these intrinsic couplings could be due to the experimental variable [10], for example, in this study, the exerted attention to the auditory stimulus delivered. This is due to the fact that the participants were not required to carry out any other tasks other than attending to the sound stimulus. Other than that, this preferable cortical network is in good agreement with previous findings that modulation of forward connection resulted in a better model than those of backward connection [26, 34, 35]. In bilinear DCM, experimentally controlled inputs and interaction between neuronal activities in a region could result in modulation of effective connectivity [13]. Thus, in the present study, we were able to show that in the context of auditory stimulus processing, the modulation has been accounted for sufficiently by the activity of the neuronal population in the bilateral STG to the intrinsic connections from each STG to both ipsilateral and contralateral HG.

Model 1 also has the element of modulation on the region itself, which is the right and left STG. We treated this modulation as stimulus-independent, and that attention has exerted directly on the neuronal population. Bayesian comparison seemed to prefer modulation effects on the STG rather than HG, which may be due to the role of the region in complex sound processing.

### Non-linear dynamic causal models

Since the neuronal origin of the modulatory influence is not specified in bilinear causal models, non-linear interaction has to be considered and the bilinear state equation needs to be modified. In non-linear causal models, matrix ***D*^(*j*)^** is included. It encodes how a particular region influences specific connections that exist in a cortical network system. This non-linear interaction is explained by the non-linear differential equation [12]:

(2) $$\frac{dx}{dt}=\left(A+\sum_{i=1}^{m}u_{i}B^{\left(i\right)}+\sum_{j=1}^{n}x_{j}D^{\left(j\right)}\right)x+Cu$$

Previously discussed models were of bilinear type and these have been used in many functional neuroimaging studies [9, 11, 36, 37]. The drawback of these models is that they assumed that the multiple inputs to a region are linearly separable [34]. Thus, they prohibit activity-dependent connections that are expressed in only one sensorimotor, attentional, or cognitive context but not in another [34]. However, non-linear networks include interactions among inputs, which is explained as an activity-dependent modulation on particular intrinsic connection. These intrinsic connections are due to the activity in other involved brain regions. Thus, a set of non-linear DCMs were constructed to determine the most probable interacting brain network in a white noise listening task. The purpose of this step was to determine whether bilinear and non-linear modulatory processes could be distinguished reliably in fMRI data. From the BMS comparison between 12 models comprising of six bilinear (Figure 5) and six non-linear models (Figure 6), Model 10, which has non-linear connectivity, was chosen as the most probable model. Model 10 has direct effect of attention on the bilateral STG, whose activity then increased the gain of STG → bilateral HG connections.

One can distinguish linear and non-linear mechanisms and assess the usefulness and validity of each of them. The critical point is whether the attention enhancement of activity in the bilateral HG would be better explained by a non-linear mechanism. In this case, is it possible that the activity in HG influenced the forward connections from each STG? Thus, the bilinear Model 1 and non-linear model Model 10 were compared with each other using Bayesian approach. Model 10 was found to be superior and resulted in <*r*> and *ϕ* of 0.9165 and 0.9995, respectively. We can say that the strength of STG to bilateral HG connection depends on the activity in the STG itself.

The non-linear nature of networks among cortical brain areas shows that the effective connectivity between them is dynamic, and the bilinear differential equation is not inherent in explaining the causality of the neuronal network [36]. From model comparison using BMS (Figure 7(a)), the relatively high difference in expected posterior and exceedance probability values between both winning linear and non-linear models can be explained by several factors. Firstly, at the level of single neurons, the fast changes in connectivity are mediated by non-linear effects, which are instances of short-term synaptic plasticity [12]. This mechanism alters the strengths with small time constants and includes a range of processes that are involved in the synaptic transmission, including facilitation, potentiation, and augmentation [37]. The history of prior synaptic activity drives these processes, thus they are non-linear [37].

Non-linear model (Figure 7(b)) is preferred because it is more accurate in terms of non-linear system identification theory, encapsulating both linear and non-linear interactions [36]. This makes the model more realistic and less based on assumption [36]. Moreover, non-linear models include interactions among inputs [34]. These interactions can be interpreted as an activity-dependent modulation of the influence that one region exerts over another, from which that activity is initiated by activity in other brain regions that exert modulatory effects [34]. Thus, non-linear models are necessary for a more suitable characterisation of contextual changes in the effective connectivity.

## Conclusion

We have shown that the brain BOLD response can be acquired using fMRI and interpreted using SPM. Group results showed that dominant right-sided brain activation is observed during white noise listening tasks, especially in the primary auditory cortex. Brain activation also presents in other regions, indicating the ability of white noise to evoke large activation areas. The neuronal network pattern comprising the activated brain regions in response to a performed task can be studied using DCM. The strength and direction of the connection can be estimated, in addition to determining the input centre and the site of modulation.

In this study, using fMRI and an analysis of effective connectivity using DCM, we found that forward modulatory influences on STG to bilateral HG couplings are augmented by exerted influence on activity in the ipsilateral STG. Changes in strength of the intrinsic connections are due to the change in activity in different regions. It was found that non-linear mechanisms can explain measured fMRI responses better than linear ones.

From this auditory fMRI study, it can be further summarised that during a binaural listening task to a white noise stimulus, the STG would act as the input centre, triggering the intrinsic connection from the STG of each hemisphere to the ipsi- and contralateral HG. Other than that, the STG region would also act to influence or augment the gain in the existing connections. However, this report only addressed the cortical network interaction in the PAC. More research needs to be done in order to study the connectivity network in further brain regions.
